# Venom Ontogeny in the Mexican Lance-Headed Rattlesnake (*Crotalus polystictus*)

**DOI:** 10.3390/toxins10070271

**Published:** 2018-07-03

**Authors:** Stephen P. Mackessy, Jamie Leroy, Estrella Mociño-Deloya, Kirk Setser, Robert W. Bryson, Anthony J. Saviola

**Affiliations:** 1 School of Biological Sciences, University of Northern Colorado, Greeley, CO 80639, USA; leroy_jk@yahoo.com (J.L.); Anthony.Saviola@med.uvm.edu (A.J.S.); 2 Departamento de Zoología, Universidad de Granada, 18071 Granada, Spain; allertsemoci@yahoo.com (E.M.-D.); kwsetser@gmail.com (K.S.); 3 Department of Biology, University of Washington, Seattle, WA 98195-1800, USA; brysonjr.rob@gmail.com; 4 Burke Museum of Natural History and Culture, Seattle, WA 98195, USA; 5 Moore Laboratory of Zoology, Occidental College, Los Angeles, CA 90041, USA; 6 Department of Molecular Medicine and Neurobiology, The Scripps Research Institute, La Jolla, CA 92037, USA

**Keywords:** bradykinin-inhibitory peptide, enzyme, evolution, phenotypic variation, toxins, venomics

## Abstract

As trophic adaptations, rattlesnake venoms can vary in composition depending on several intrinsic and extrinsic factors. Ontogenetic changes in venom composition have been documented for numerous species, but little is known of the potential age-related changes in many rattlesnake species found in México. In the current study, venom samples collected from adult and neonate *Crotalus polystictus* from Estado de México were subjected to enzymatic and electrophoretic analyses, toxicity assays (LD_50_), and MALDI-TOF mass spectrometry, and a pooled sample of adult venom was analyzed by shotgun proteomics. Electrophoretic profiles of adult males and females were quite similar, and only minor sex-based variation was noted. However, distinct differences were observed between venoms from adult females and their neonate offspring. Several prominent bands, including P-I and P-III snake venom metalloproteinases (SVMPs) and disintegrins (confirmed by MS/MS) were present in adult venoms and absent/greatly reduced in neonate venoms. Age-dependent differences in SVMP, kallikrein-like, phospholipase A_2_ (PLA_2_), and L-amino acid oxidase (LAAO) activity levels were confirmed by enzymatic activity assays, and like many other rattlesnake species, venoms from adult snakes have higher SVMP activity than neonate venoms. Conversely, PLA_2_ activity was approximately 2.5 × greater in venoms from neonates, likely contributing to the increased toxicity (neonate venom LD_50_ = 4.5 μg/g) towards non-Swiss albino mice when compared to adult venoms (LD_50_ = 5.5 μg/g). Thrombin-like (TLE) and phosphodiesterase activities did not vary significantly with age. A significant effect of sex (between adult male and adult female venoms) was also observed for SVMP, TLE, and LAAO activities. Analysis of pooled adult venom by LC-MS/MS identified 14 toxin protein families, dominated by bradykinin-inhibitory peptides, SVMPs (P-I, P-II and P-III), disintegrins, PLA_2_s, C-type-lectins, CRiSPs, serine proteinases, and LAAOs (96% of total venom proteins). Neonate and adult *C. polystictus* in this population consume almost exclusively mammals, suggesting that age-based differences in composition are related to physical differences in prey (e.g., surface-to-volume ratio differences) rather than taxonomic differences between prey. Venoms from adult *C. polystictus* fit a Type I pattern (high SVMP activity, lower toxicity), which is characteristic of many larger-bodied rattlesnakes of North America.

## 1. Introduction

The rattlesnakes *Crotalus* and *Sistrurus* comprise a monophyletic lineage of vipers unique to the Americas, and they are easily recognized by the presence of the caudal rattle. Rattlesnakes appear to be most closely related to the New World pitvipers, with American *Agkistrodon* being their sister group [1]. It has been hypothesized that rattlesnakes originated in México [2,3], diversifying and dispersing to the north and south, and they have a current range extending over 70 degrees of latitude, from southern Canada to central Argentina and southern Uruguay [4]. However, even with this wide geographical distribution, the highest diversity of rattlesnakes occurs on the Central Mexican Plateau and surrounding highlands [4]. Despite this diversity, venoms of most Mexican rattlesnakes, particularly the montane species, are poorly known. Exceptions include species that represent the greatest risks to humans, such as *C. simus* [5,6] and *C. scutulatus scutulatus* [7]. Several recent studies have also examined the venom compositional patterns of the diminutive *C. lepidus* [8,9,10] and *C. willardi* [10] subspecies. 

Snake venoms are complex secretions of proteins and peptides produced in specialized cephalic glands [11,12]. The venom from a single individual may contain up to 100 proteins, including isoforms, although most venom compounds can be classified into 10–15 protein families [13,14]. For rattlesnakes these families are predominately the enzymatic L-amino acid oxidases (LAAO), phosphodiesterases (PDE), metalloproteinases (SVMP), serine proteases (SVSP), and phospholipases A_2_ (PLA_2_), and the non-enzymatic myotoxin a homologs, disintegrins, cysteine-rich secretory proteins (CRiSPs), and C-type lectins [15,16]. Rattlesnake venoms have been shown to vary in composition as a function of species [13,15,17], age [18,19,20,21,22,23,24,25,26,27], and geography [10,28,29,30]. Several other factors, such as season and sex, may also affect individual venom composition [15,16]. However, of all identified factors, phylogeny and ontogeny appear to have the most profound influences on venom phenotypes. 

**Figure 1:**
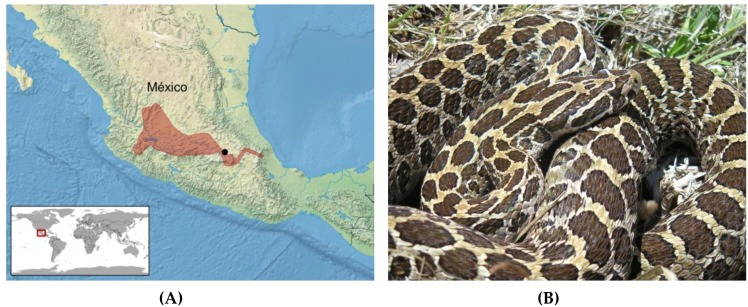
(**A**) Distribution of *Crotalus polystictus* (red) in México (after Campbell and Lamar [4]). The approximate location of México City is shown by the black dot. (**B**) Adult *C. polystictus*; photo by EMD.

Snakes are gape-limited predators that often show distinct, age-related changes in prey preferences likely associated with prey availability, handling capacity or both [31]. For many species, this change includes a shift from consuming lizards and small/neonate rodents by juvenile snakes, to taking larger rodents as adults [2,21]. This dietary shift often coincides with an age-related shift in venom composition, as has been demonstrated for several northern latitude species and several clades of Central and South American rattlesnakes [20,21,25,26,27,32]. However, to date, little is known of the venom ontogeny patterns among Mexican rattlesnake species. A long-term ecological study of the Mexican Lance-headed Rattlesnake (*Crotalus polystictus*) in Estado de México [33,34,35] afforded the opportunity to obtain venom samples from neonate, juvenile, and adult rattlesnakes. This medium-sized species averages 600–700 mm in length and occupies native and modified grasslands in central México, and is a mid-elevation species, occurring from approximately 1450–2600 m ([4]; Figure 1). In this population, all age classes appear to feed primarily on rodents, with neonates gape-limited to utilizing only smaller species and individuals (Mociño-Deloya et al., in prep). Following the type I (high SVMP activity and low toxicity, >1.0 mg/g mouse body weight) and type II (low SVMP activity and high toxicity, <1.0 mg/g mouse body weight) classification put forth by Mackessy [15,16], adult *C. polystictus* possess a type I venom [15]. Transitions from a type-II-like venom in neonates to a type I venom in adults are observed for several rattlesnake species found in the United States [15,21], and therefore it was hypothesized that *C. polystictus* would also show prominent venom ontogenetic changes in composition. We report here results of a detailed analysis of ontogenetic changes in venom composition, sex-related differences in toxin activities, and LC-MS-MS analysis of the venom proteome in this Mexican rattlesnake.

## 2. Results

### 2.1. Enzyme Analyses

**Figure 2:**
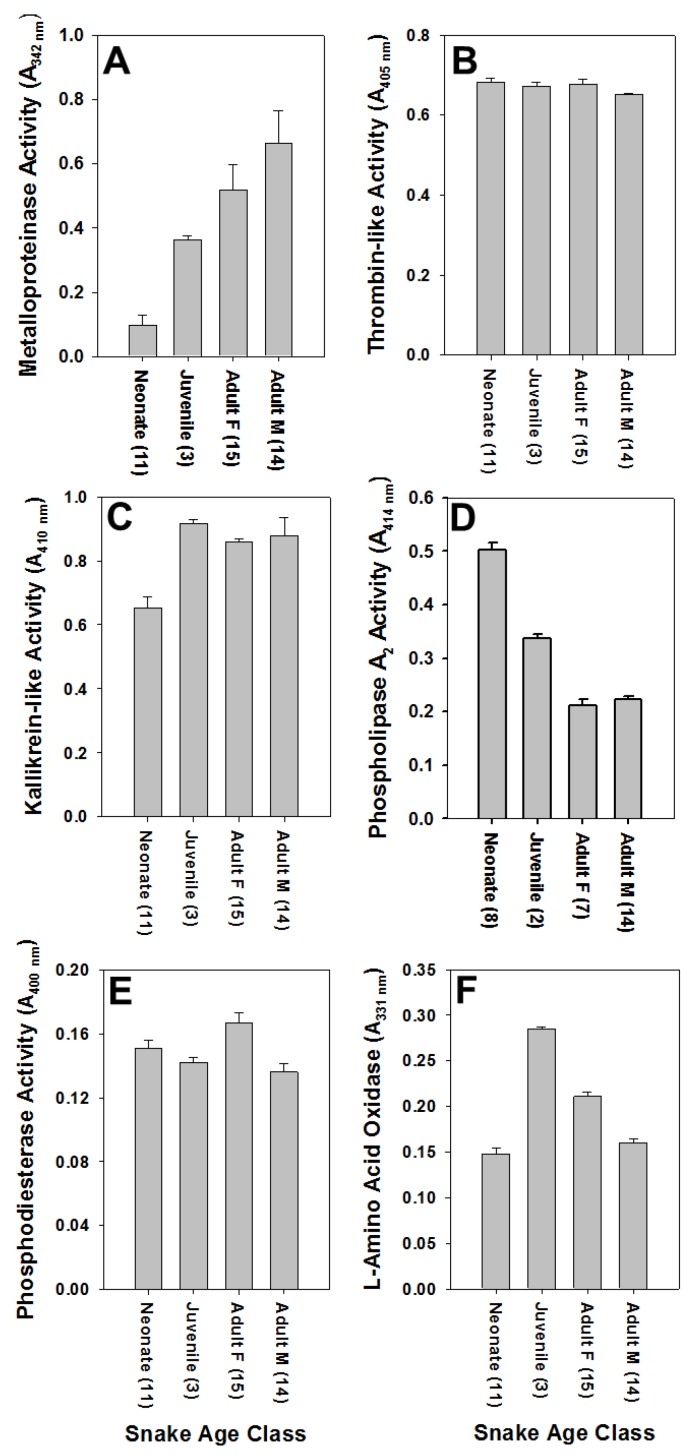
Enzyme activities in *Crotalus polystictus* venoms as a function of age class and sex; sample size for each is given parenthetically. Note that metalloproteinases (SVMP) activity **(A)** is lowest and PLA_2_ activity **(D)** is highest in neonate venoms; adult venoms show the opposite trend. Values are shown as averages ± SD. (**A)** Metalloproteinase activity; (**B)** Thrombin-like activity; (**C)** Kallikrein-like activity; (**D)** Phospholipase A_2_ activity; (**E)** Phosphodiesterase activity; (**F)** L-amino acid oxidase activity.

**Figure 3:**
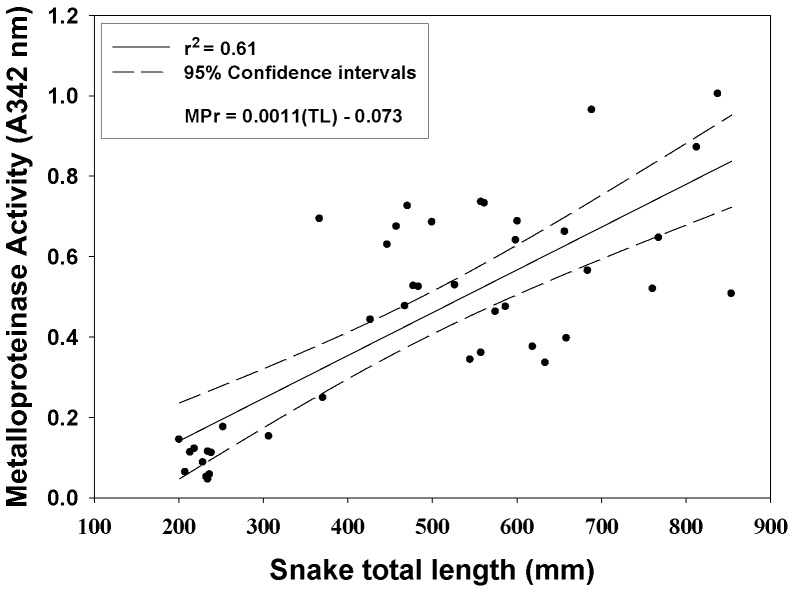
SVMP activity of *C. polystictus* venoms as a function of total snake length; a significant ontogenetic effect is apparent. *n* = 43.

All *C. polystictus* venoms exhibited activities of six enzymes commonly found in rattlesnake venoms (Figure 2). Metalloproteinases activity showed a statistically significant difference with age (Figure 3; F_2,40_ = 37.94, *p* < 0.001), typical of many rattlesnake venoms [21]; average activity of adult venoms was approximately 6.5 × that of neonate venoms (*p* < 0.001). Juvenile and neonate venoms were also significantly different for SVMP activity (*p* < 0.05), whereas a comparison of juvenile and adult venoms was not significantly different (*p* = 0.065). Kallikrein-like activity differences were statistically significant (F_2,40_ = 14.28, *p* < 0.001), with neonates also having significantly less activity compared to both juvenile and adult age classes (both *p*’s *<* 0.01); juvenile and adult activity differences were not significant (*p* = 0.795). Phospholipase A_2_ activity showed an inverse relationship with age that was statistically significant (F_2,29_ = 41.42, *p* < 0.001), and activity in neonate venoms was 2.5 × greater than in adult venoms (*p* < 0.001). A comparison of juvenile and neonate PLA_2_ activity also showed significant differences (*p* < 0.01); differences in juvenile and adult activity was again not statistically significant (*p* = 0.083). A statistically significant difference in relation to age class was observed as well for LAAO activity (F_2,39_ = 3.894, *p* < 0.05), with a significant difference being observed between neonate and juvenile age groups (*p* < 0.05). TLE and PDE activities did not show statistically significant differences for any age classes (all *p*’s > 0.05). 

Metalloproteinases, TLE, and LAAO activities also showed a significant effect of sex (Student’s *t*-test). Venoms from adult male snakes exhibited significantly higher SVMP activity than venoms of adult female snakes (*t* = 2.5057, df = 27, *p* < 0.05), whereas adult females had significantly higher TLE (*t* = 3.6904, df = 27, *p* < 0.01) and LAAO (*t* = 2.1026, df = 27, *p* < 0.05) activities than venoms of adult male snakes. There was no apparent effect of sex on kallikrein-like, PDE, or PLA_2_ activities (all *p*’s > 0.05).

### 2.2. 1-D SDS-PAGE

**Figure 4:**
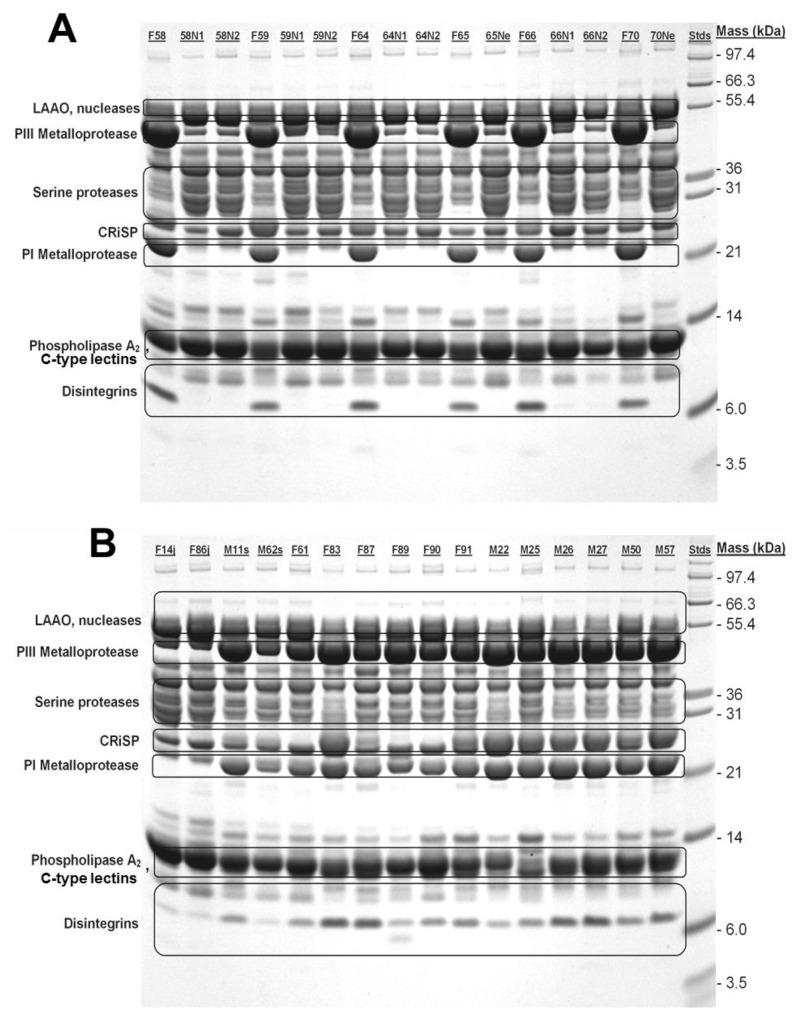
SDS-PAGE analysis of neonate, juvenile, and adult *C. polystictus* venoms. (**A**) Adult female and neonate venoms. (**B**) Juvenile, subadult and adult venoms. While most bands are shared between samples, note the absence of P-I SVMP and disintegrin bands from neonate venoms; P-III SVMP are also greatly reduced in intensity in neonate venoms. Identical numbers (i.e., F58 and 58n1) indicate venoms from a female and her neonate offspring. Abbreviations: F, female; M, male; N, neonate; j, juvenile.

Typical of many venoms from within a species, *C. polystictus* venoms shared most components as revealed by 1D SDS-PAGE (Figure 4). Enzymatic LAAOs, nucleases (PDEs), SVSPs, and PLA_2_, in addition to the non-enzymatic CRiSP and C-type lectins appear in all individuals examined. Electrophoretic analysis demonstrated that at least two subtypes of SVMPs, P-I and P-III [36,37], vary ontogenetically, and these components were much more prevalent (high band density) in adult snake venoms. These results are consistent with enzyme data reported above. In addition, a low-mass component (approx. 7 kDa), putatively a disintegrin, appears to be absent from neonate venoms, faint in juvenile venoms, and prominent in venoms of adult snakes.

### 2.3. MALDI-TOF-MS Mass Fingerprinting of Venoms

**Figure 5:**
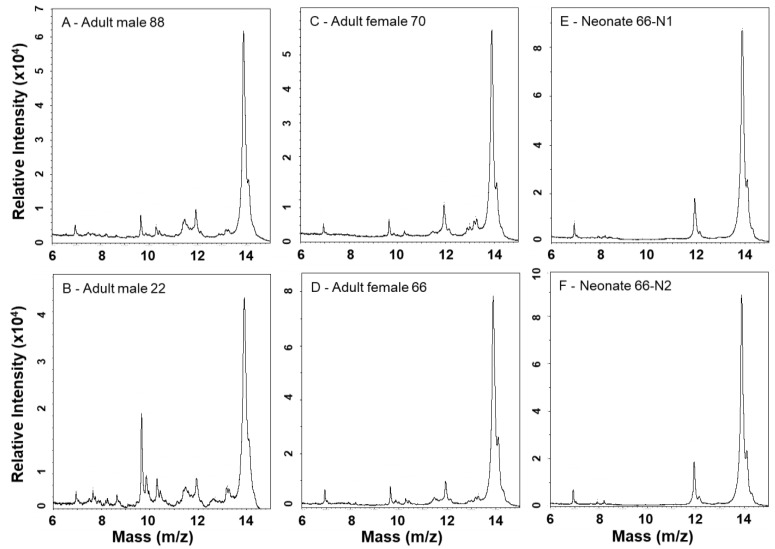
MALDI-TOF-MS analysis of *C. polystictus* venoms; 6–15 kDa mass window.

**Figure 6:**
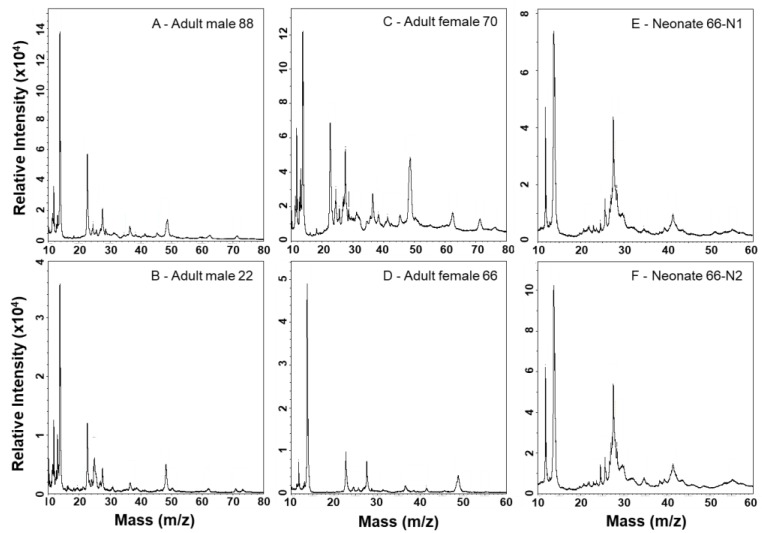
MALDI-TOF-MS analysis of *C. polystictus* venoms; 10–80 kDa mass window.

MALDI-TOF mass spectrometry provides more detailed analyses of lower mass components and is complementary to SDS-PAGE (Figure 5 and Figure 6). A PLA_2_ of 13,905 Da dominates all spectra, and numerous other small peaks are seen between approx. 6.9–11.9 kDa; the prominence of these lower mass components appears muted in neonate venoms, suggesting a lower abundance of these proteins as also observed by SDS-PAGE. Adult venoms showed prominent peaks at approx. 22.6 kDa (likely a P-I SVMP) that were absent from neonate venoms (Figure 6), again consistent with the enzymatic activity assay and SDS-PAGE data. Clusters of several peaks in the 24–30 kDa range are consistent with masses seen for CRiSPs and SVSPs, and these were variable in overall intensity between samples. In general, neonate venoms appeared to contain fewer peaks than adult venoms.

### 2.4. MALDI-TOF-MS/MS Identification of Specific Proteins

**Figure 7:**
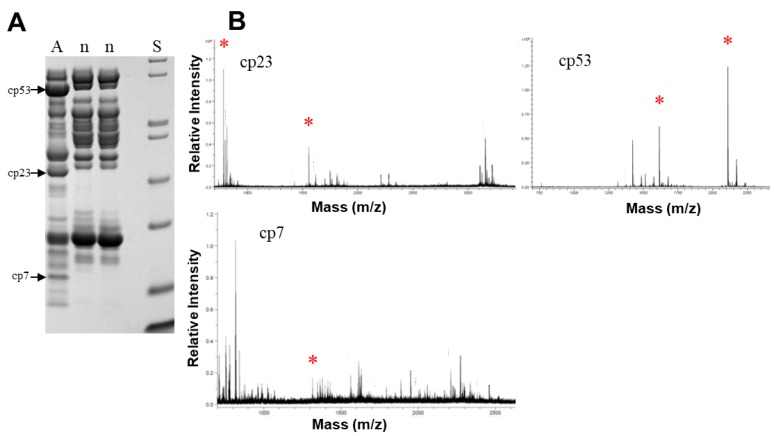
MALDI-TOF-MS/MS identification of several venom proteins only expressed in adult venoms of *C. polystictus*. (**A**) SDS-PAGE showing three bands excised from gel. (**B**) Bands indicated were identified as a PIII SVMP (cp53), a P-I SVMP (cp23), and a SVMP degradation fragment (cp7). A, adult female; n, two different neonates; S, mass standards. *, indicates peptides providing positive ID.

Identity of three venom proteins that varied with age class (SDS-PAGE) was confirmed by MALDI-TOF-MS/MS (Figure 7). These proteins were present in the adult female venom but were absent from venoms of her neonate offspring. Positive hits for the protein band at 53 kDa in adult venom were obtained: peptide 1 (mass = 2110.09; ITVKPEADYTLNAFGEWR) showed identity with a P-III SVMP (T1E6U1) from *Crotalus oreganus helleri* venom. Shotgun proteomic analysis (see below) also identified this peptide at m/z 1055.53 (2^+^) and m/z 704.02 (3^+^) (Table S1). Peptide 2 had a mass of 1615.81, and yielded the sequence MYELANTVNEIYR matching to a P-III SVMP (Q2QA02) from *Crotalus d. durissus* venom. This peptide was also identified at m/z 808.39 (2^+^). Similarly, two peptides from the digest of the 23 kDa band showed positive hits: peptide 1 (mass = 1554.79; STGVVQDHSEINLR) showed identity with a P-II SVMP from *Crotalus atrox* venom (atrolysin E; (P34182)). This peptide was also identified at m/z 777.90 (2^+^), 518.94 (3^+^), 570.97 (3^+^), and 428.48 (4^+^), with the latter two ions containing an additional N-terminal arginine accounting for the mass difference of 156.1 (Table S1). Peptide 2 (mass = 781.37; SFGEWR) also showed identity with atrolysin E. It should be noted that atrolysin E is a P-II SVMP that is processed to an active proteinase and a disintegrin-like protein [38,39], and therefore, an estimated mass of 23 kDa (by SDS-PAGE) is consistent with this enzyme in its active form. 

The 7 kDa band yielded a peptide (mass = 1313.79; RYIELVVVADHRV) which showed identity with several *Crotalus* venom SVMPs (Q90392.1, Q90391.1, P15167.3, all from *C. atrox*). This band, which was also observed via shotgun proteomics (below), appears to be a degradation fragment of a larger SVMP. For all protein hits, MASCOT identity scores of ≥16 were obtained, strongly supporting identity (*p* < 0.05) with the fragment regions of the respective proteins.

### 2.5. Mass Spectroscopy: Orbitrap LC-MS/MS Identification of Venom Proteome

**Figure 8:**
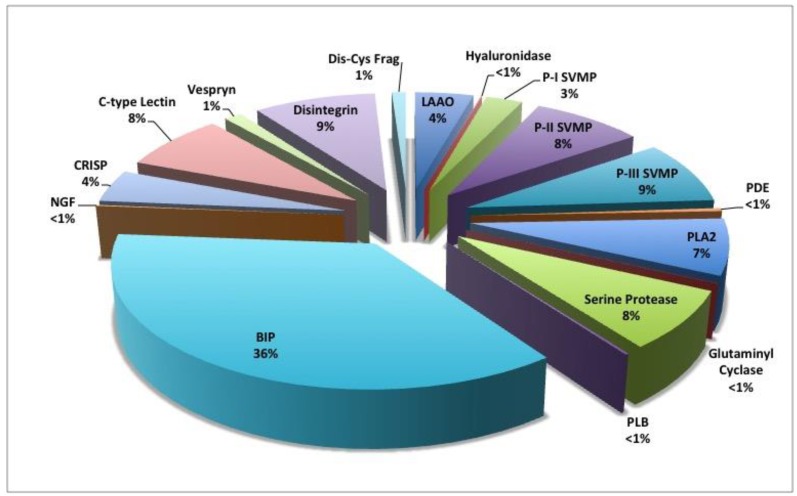
Venom proteome of adult *C. polystictus*. Abbreviations: BIP, bradykinin-inhibitory peptide; CRiSP, cysteine-rich secretory protein (helveprin); Dis-Cys Frag, cysteine-containing disintegrin fragment; LAAO, L-amino acid oxidase; NGF, nerve growth factor; PDE, phosphodiesterase (exonuclease); PLA2, phospholipase A_2_; PLB, phospholipase B; P-I SVMP, class P-I snake venom metalloproteinase; P-II SVMP, class P-II snake venom metalloproteinase; P-III SVMP, class P-III snake venom metalloproteinase.

Shotgun proteomic analysis of *C. polystictus* venom identified 14 protein families (Table 1 and Figure 8; Table S1); relative abundance (% of total proteins) was based on normalized spectral abundance factor (NSAF), which takes into account both spectral counts and parent protein mass. The peptide TPPAGPDVGPR, which is identical to a bradykinin-inhibitory peptide (P0CJ34) accounts for over 36% of the total venom proteome. Consistent with other type I venoms, *C. polystictus* venom exhibits moderate SVMP levels, (~19%) representing all three SVMP subfamilies: P-I—2.7%, P-II—8.2%, and P-III—8.6%. Serine proteinases comprise 7.8% of the entire venom proteome, with thrombin-like (5.7%), kallikrein-like (1.9%), and a plasminogen activator (T1D6M5) (0.25%) all being identified by proteomic analysis. Basic (6.0%) and acidic (1.5%) PLA_2_s, and LAAOs (4.4%) were present at moderate levels. The non-enzymatic disintegrins, C-type lectins, and CRiSPs accounted for approximately 9.0%, 7.8%, and 4.4% of the entire venom proteome, respectively. The remaining seven protein families accounted for a total of approximately 4% of venom proteins.

### 2.6. Toxicity of Neonate and Adult Venom to Mice

**Figure 9:**
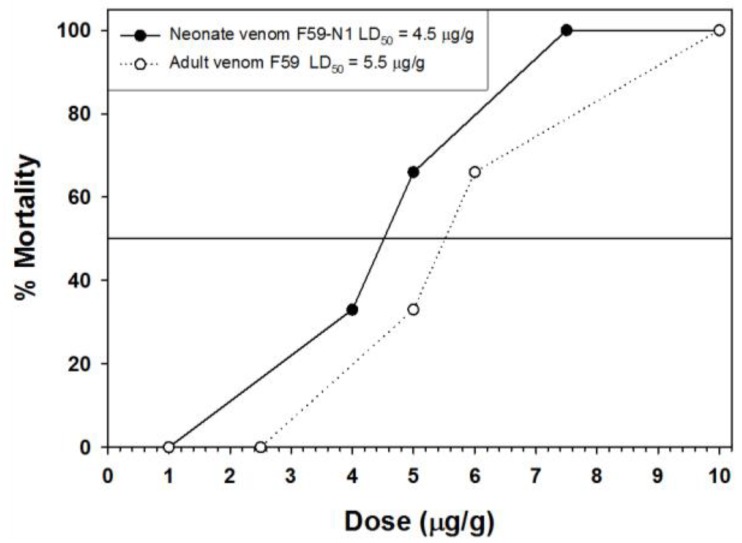
Lethal toxicity (24 h post-injection) of *C. polystictus* venoms toward NSA mice.

As shown in Figure 9, venom from neonate snakes was somewhat more toxic to NSA mice (LD_50_ = 4.5 μg/g) than adult venom (LD_50_ = 5.5 μg/g; Figure 9), a pattern typically observed with type I venoms. 

## 3. Discussion

As trophic adaptations, snake venoms have allowed for the transition from a mechanical (constriction) to a chemical (venom) means of incapacitating and killing prey. Ontogenetic shifts in venom enzymatic and toxic activities often coincide with shifts in prey preference, and the general shift from low SVMP activity and high toxicity in neonates, to high SVMP activity and low toxicity in adult rattlesnakes is frequently observed. The increased SVMP content in adult rattlesnakes likely facilitates tissue degradation of larger, more metabolically favorable prey items, such as rodents. However, the opposite relationship has also been documented in some populations of *C. viridis viridis* [26], in which adult venoms have significantly lower SVMP but higher myotoxin a levels than neonates. The increased concentration of myotoxin a is in support of an increase in lethal toxicity towards mammals, as adult *C. v. viridis* consume almost entirely small mammals and this toxin is specific toward rodents [40]. In addition, paedomorphic trends in venom compositional patterns, where adults retain juvenile venom characteristics, have also been documented in *C. o. concolor* [24], *C. simus*, and *C. durissus* [25].

Like the venoms of several temperate species of rattlesnakes, venoms from *C. polystictus* undergo prominent shifts in composition during postnatal development. Most striking is the change in SVMP activity, which in adult snake venoms occurs via increased expression of at least two classes of SVMPs (P-I and P-III) as detected by SDS-PAGE and MALDI-TOF MS/MS. Proteomic analysis further confirmed the presence of all three subfamilies of SVMPs (P-I, P-II, and P-III) in pooled adult *C. polystictus* venom. This compositional pattern appears to be common among rattlesnakes producing type I venoms. In *C. o. helleri* and *C. o. oreganus* [21,23], this shift is correlated with a change in diet, from lizards and neonate rodents to larger mammalian prey; venoms from these taxa also show a significant increase in thrombin-like serine protease activity (unpubl. data). However, *C. polystictus* in this population feeds primarily on small mammals (mammals comprised 87.9% of 545 prey items) throughout their ontogeny, taking juvenile rodents (pygmy mice, *Baiomys taylori*, and shrews) when neonates and expanding their diet to include larger rodents, especially *Microtus mexicanus*, as adults (Mociño-Deloya et al., in prep.). This suggests that the main impetus for increases in SVMP activities is utilization of larger, bulkier prey, as has been previously suggested [21], rather than a shift to different taxa of prey. This conclusion is further supported by the fact that adult male *C. polystictus* consume a greater proportion of larger mammalian prey compared to adult female *C. polystictus*; venoms from adult males had significantly higher SVMP activity compared to the venoms from adult females, perhaps reflecting the importance of larger rodents and lagomorphs in the diet of adult males compared to females (24.0% vs. 5.1% of prey mass) [35].

As was also observed with *C. o. oreganus* and *C. o. helleri* venoms, neonate *C. polystictus* venoms contained over 2× the levels of PLA_2_ activity seen in venoms of adult snakes. In *Crotalus simus simus*, this differential was found manifested as the presence of crotoxin homologs in neonate venoms [41]. However, in *C. polystictus*, all dominant venom PLA_2_s showed identical masses of 13,906 Da by MALDI-TOF MS, strongly indicating that a crotoxin homolog is *not* present in these venoms. Shotgun proteomic analysis also failed to identify crotoxin or Mojave toxin in *C. polystictus* venom, although several acidic and basic PLA_2_s are clearly present in the pooled sample. Most notable are peptides that matched a PLA_2_ (A0A0K8RYR4) from *C. horridus*, which comprises approximately 5% of the adult *C. polystictus* venom proteome. The lack of crotoxin is also supported by lethality assays in mice, as rattlesnake venoms containing crotoxin homologs show lethal toxicities below 1.0 µg/g; venoms from neonate *C. polystictus* are considerably less toxic. The slight increase observed with neonate venom toxicity, as compared to adults, may compensate for the significantly smaller venom bolus that can be injected into prey during a predatory strike, and a more toxic venom enables neonate snakes to dispatch prey items more rapidly. However, the similarity in toxicity seen in *C. polystictus* venoms may also be a reflection of their mammals-only diet; in *C. oreganus helleri* and *C. o. oreganus*, juvenile venoms were approximately 2× as toxic as adult venoms, and this species utilizes lizards as a dominant prey of neonate and young snakes [21].

Shotgun proteomic analysis indicates that the venom proteome of adult *C. polystictus* comprises just over 9% disintegrins, and SDS-PAGE analysis shows a clear distinction in disintegrin band intensity between adult and neonate venoms. Disintegrins are small (4–16 kDa) non-enzymatic proteins that often result from a post-translational processing of the P-II class of SVMPs [42,43,44]. Therefore, the lack of a disintegrin band in neonate venoms may be correlated with the lower SVMP content and activity observed in these venoms. By blocking integrin α_IIb_β_3_, disintegrins inhibit platelet aggregation and facilitate the circulation of toxins throughout prey [45]. In *C. atrox*, and likely other species, disintegrins also appear to act as a molecular “tag” by altering the chemical scent of prey and allowing for successful prey recovery by strike-induced chemosensory searching [46]. For adult *C. polystictus,* the increased disintegrin concentration may assist with successful prey relocation during predatory episodes, as adults frequently release rodent prey following the envenomating strike. Neonates, on the other hand, often strike-and-hold prey, and therefore an abundance of disintegrin(s) in their venom is not necessary. The lack of disintegrins in neonate *C. polystictus* venom is also likely compensated by the increased toxicity, as venom would more rapidly debilitate prey before it retaliated or could wander far from the attack site. The concentration of disintegrin required for successful prey recovery remains unknown. However, in *C. o. concolor*, which exhibits a type II venom phenotype [24] and has significantly less disintegrin compared to type I species such as *C. atrox*, *C. o. oreganus/helleri* and *C. polystictus*, successful discrimination between envenomated and non-envenomated prey cues has been documented [47].

In addition to the marked ontogenetic shift discussed above, significant age-related differences were also observed for kallikrein-like and LAAO activities, with neonate venoms having significantly lower activity levels for both enzymes. On the other hand, there does not appear to be a significant age-dependent trend for TLE and PDE activities, although TLE and LAAO activities did demonstrate a sex-based difference in adult snakes; adult females had statistically higher activity levels when compared to adult males, but overall levels were similar between age classes. This lack of age-related variation is consistent with a mammals-only diet, as TLE serine proteinases are likely more effective toward mammals, which generally have much narrower tolerances than reptiles for variation in blood pressure and clotting. TLE and kallikrein-like serine proteases target the blood-coagulation cascade, and TLEs have received significant attention for their ability to cleave fibrinogen and deplete the major clotting factors that would normally assist with clot formation [22,23,48,49,50]. Phosphodiesterases, which comprises <1% of the *C. polystictus* venom proteome, generates purine nucleosides and may assist with prey immobilization via hypotension [51,52]. L-amino acid oxidases, which make up approximately 4% of the venom proteome, appear to have multiple roles in subduing prey. L-amino acid oxidase activity likely contributes to overall toxicity via production of hydrogen peroxide during the enzymatic deamidation of amino acids [53], induction and inhibition of platelet aggregation [54,55], hemorrhage [56] and cell apoptosis [55,57,58]. Examination of SDS-PAGE electrophoretic patterns showed that neonate, juvenile, and adult venoms shared most other major venom protein components.

The major toxin present in adult *C. polystictus* venom was the bradykinin-inhibitory peptide (BIP) TPPAGPDVGPR [P0CJ34], which accounts for 36% of the venom proteome using NSAF. Bradykinin-inhibitory peptides result from the proteolytic processing of a larger (approx. 180 residue) bradykinin-potentiating peptide (BPP)/C-type natriuretic peptide (CNP) precursor [59], and they are broadly distributed in the venoms of New World pit vipers [25,26,59,60,61,62], albeit at significantly lower concentrations than in *C. polystictus* venom. Bradykinin-inhibitory peptides appear to antagonize the vasodilatory actions of bradykinin at the B2 receptor, suggesting that they may disrupt normal cardiovascular function and enhance the overall toxicity of venom [59]. The high abundance of this peptide in *C. polystictus* venom suggests that they likely play a pivotal role in the action of snake venoms. Interestingly, Pla et al. [63] identified 12 BPP-like peptides, accounting for 28% of the total venom proteome of *Lachesis muta rhombeata*; however, an incredibly high dose of pooled BPP-like peptides had no effect on mice following intravenous and intraperitoneal administration, so their role in envenomation remains obscure.

In addition to the venom components discussed above, shotgun proteomic analysis of *C. polystictus* venom identified various proteins of lesser abundance (≤1% each), including glutaminyl cyclase (M9NCD3), phospholipase B (PLB) (A0A077L7E7), vespryn (F8S122), a disintegrin-cysteine fragment (E9JG43), hyaluronidase (U3TBU1), and a nerve-growth factor (T1DEA2). It should be noted, however, that our analysis identified only a single peptide matching to hyaluronidase (U3TBU1) and nerve-growth factor (T1DEA2) (Table S1). Although present in low concentrations in snake venoms, glutaminyl cyclases may play a significant role in post-translational modifications by enzymatically converting the N-terminal formation of pyroglutamate of several snake venom protein families [64,65]. This may stabilize these compounds and therefore contribute to overall venom toxicity. Glutaminyl cyclases may also be involved in the biosynthesis of pyroglutamyl peptides such as BPPs [66,67] and tripeptide inhibitors of SVMPs [68]. Phospholipase B has been correlated with high direct hemolytic activity of venoms from several Australian elapids [69,70,71]; however, it remains unknown if they have similar roles in viperid venoms. Hyaluronidase increases venom potency and degrades local tissue by breaking down hyaluronic acid in the extracellular matrix and facilitating the diffusion of additional venom compounds throughout prey [72]. Vespryn and nerve-growth factors, although becoming more frequently identified in venom, have elusive biological roles. Additionally, many trace components are detected in venoms by mass spectrometry, but whether they have any role during envenomation seems doubtful, as most are of low toxicity and their effects, if any, would be greatly overshadowed by more prominent toxins.

## 4. Conclusions

Venom ontogeny has been widely documented in viperid [21,24,25,26,73,74,75,76,77], elapid [78,79,80], and several rear-fanged “colubrid” [81,82] snakes, and it appears to be largely driven by natural selection for different diets [21,83]. *Crotalus polystictus* shows clear distinction between neonate and adult venoms, with SVMP, kallikrein-like, LAAO activities, and disintegrin content being significantly higher in adult venoms, whereas neonates exhibit higher PLA_2_ activity. Surprisingly, with adult venoms, there is also a significant sex-based difference in SVMP, LAAO, and TLE activities. Although both neonate and adult *C. polystictus* exhibit a type I venom phenotype, the differences in venom composition are likely driven by prey size rather than shifts in prey type, as both neonate and adult *C. polystictus* consume rodent prey. Results of this and other studies indicate that there are several variations in the basic patterns of rattlesnake venom ontogeny, suggesting that venom ontogeny, like venom compositional variation generally, is shaped by multifactorial determinants.

## 5. Materials and Methods 

### 5.1. Reagents

Invitrogen NuPage 12% bis-tris electrophoretic gels, buffers, and Mark 12 molecular weight standards were obtained from ThermoFisher Scientific (Waltham, MA, USA). Protein concentration reagents were purchased from BioRad, Inc. (San Diego, CA, USA). Soluble phospholipase A_2_ assay kit (765001) was purchased from Cayman Chemical Co. (Ann Arbor, MI, USA). All other reagents used (analytical grade or better) were obtained from Sigma Chemical Corp. (St. Louis, MO, USA).

### 5.2. Venoms

Venoms from 43 snakes (adults (>450 mm SVL):15 females, 14 males; juveniles (250–325 mm):1 female, 2 males; neonates (<240 mm):6 females, 5 males) were collected in the field and stabilized via desiccation over silica gel. Venoms were then stored with desiccant at −20 °C until used. All snake handling and venom collection was conducted under permit NUM/SGPA/DGVS/06320 from México (issued to E. Mociño-Deloya). 

### 5.3. Enzyme Assays 

All venom samples were solubilized in Millipore-filtered ddH_2_O at 4 mg/mL. Before experimental use, venom samples were brought to room temperature, vortexed, and centrifuged. Protein concentrations were determined [84] using bovine γ globulin as standard, and all enzyme assays (run in triplicate) were based on these values.

Metalloproteinase (SVMP) activity was determined using azocasein as substrate [85]. Serine proteinase (SVSP) activities (thrombin-like and kallikrein-like) were assayed using p-nitroaniline tripeptide substrates [22]. Phosphodiesterase (PDE) activity was determined using Ca-bis-nitrophenyl phosphate [86,87]. L-amino acid oxidase (LAAO) assays followed methods described previously using L-kynurenine as a substrate [21]. All results are reported as product formed/min/mg of venom protein.

Phospholipase A_2_ (PLA_2_) activity was determined by a modification of the assay procedure recommended by the supplier (Cayman Chemical Co.); all samples were run in duplicate, but due to limited samples, only 31 venoms were assayed (all age classes represented). Substrate (diheptanoyl thio-phosphatidyl choline) was solubilized at 1.66 mM in assay buffer (25 mM tris-HCl, pH 7.5 with 10 mM CaCl_2_, 100 mM NaCl, and 0.3 mM Triton X-100) and diluted 1:3 with assay buffer (=working substrate solution) just before use. Ten µL assay buffer (blank), bee venom PLA_2_ (positive control) or 0.5 µg venom was added to wells of a 96-well plate and then 10 µL DTNB (10 mM) was added. Two hundred µL working substrate solution was then added to a column of assays on the plate to initiate reactions and the plate was placed in a Spectramax 190 plate reader at 37 °C; absorbance readings (414 nm) were taken every minute for 10 min. Enzyme activity was calculated from the linear portion of reaction rate curves (between 2.0 and 3.0 min), and specific activity was expressed as µmol product formed/min/mg protein.

### 5.4. One-dimensional SDS Gel Electrophoresis

Dithiothreitol (DTT) reducing sodium dodecyl sulfate polyacrylamide gel electrophoresis (SDS-PAGE) was conducted using Nu Page 12% acrylamide gels and Mark 12 standards (Life Technologies, Inc. (Grand Island, NY, USA) as described previously [88]. Twenty-four μg of venom was loaded into each lane. Gels were stained in 0.1% Coomassie brilliant blue R-250, destained with 30% methanol/7% acetic acid, and photographed using a Bio-Rad gel imaging system. Protein class was identified based on mass and published accounts [15,16].

### 5.5. Mass Spectrometry: MALDI-TOF

Two representatives each of neonate, adult female, and adult male venoms were subjected to mass fingerprinting. Approximately 0.5 μg of venom in 1.0 μL 50% acetonitrile (ACN) in ddH_2_O was mixed with 1 μL sinapinic acid matrix (10 mg/mL 50% ACN in ddH_2_O), spotted onto MALDI target plates, allowed to air dry and analyzed using an Ultraflex-TOF/TOF mass spectrometer (Bruker Daltonics, Billerica, MA, USA) in operating in linear mode using a 25 kV accelerating voltage.

### 5.6. Mass Spectrometry: Orbitrap LC-MS/MS

One pooled venom sample (3 male, 3 female, adults) was subjected to shotgun proteomic analysis (Florida State University College of Medicine Translational Science Laboratory) in order to obtain an overview of the total venom proteome of *C. polystictus*. Samples were digested using the Calbiochem ProteoExtract All-in-one Trypsin Digestion kit (Merck, Darmstadt, Germany) with LC/MS grade solvents according to the manufacturer’s instructions. The LC-MS/MS analyses were performed using an LTQ Orbitrap Velos equipped with a Nanospray Flex ion source and interfaced to an Easy nanoLC II HPLC (Thermo Scientific). Peptide fragments were separated using a vented column configuration consisting of a 0.1 × 20 mm, 3 mm C_18_ trap column, and a 0.075 × 100 mm, 3 mm C_18_ analytical column (SC001 and SC200 Easy Column respectively, Thermo Scientific). The elution gradient consisted of 5% buffer B (0.1% formic acid in HPLC grade acetonitrile), and 95% buffer A (0.1% formic acid) at the run start, to 35% B at 60 min, to 98% B from 63 to 78 min with a flow rate of 600 nL/min from 64 to 78 min, and 5% B at 300 nL/min at 79 min. The mass spectrometer was operated in positive mode nanoelectrospray with a spray voltage of + 2300 V. A “Top 9” method was used with precursor ion scans in the Orbitrap at 60 K resolving power and fragment ion scans in the linear ion trap. Precursor ion selection using MIPS was enabled for charge states of 2^+^, 3^+^ and 4^+^. Dynamic exclusion was applied for 60 s at 10 ppm. ITMS scans were performed using collision-induced dissociation (CID) at 35% normalized collision energy. MS/MS peptide spectra produced were interpreted using Mascot (Matrix Science, London, UK; version 1.4.0.288), SEQUEST (Thermo Fisher Scientific, San Jose, CA, USA; version 1.4.0.288), and X! Tandem (thegpm.org; version CYCLONE 2010.12.01.1), assuming a trypsin digestion. The Mascot5_Trembl_bony vertebrate database and the SEQUEST and X! Tandem Uniprot Serpentes (A8570) databases were used for homology searches. SEQUEST and X! Tandem were searched with a fragment ion mass tolerance set to 0.6 Da, and a parent ion tolerance of 10 ppm. Mascot was searched with a fragment ion mass tolerance of 0.8 Da, and a parent ion tolerance of 10 ppm. Glu/pyro-Glu of the N-terminus, ammonia loss of the N-terminus, carbamidomethylation of cysteines and carboxymethylation of cysteines were specified as variable posttranslational modifications within X! Tandem. Oxidations of methionine, carbamidomethyl cysteine, and carboxymethyl cysteine were specified as variable post-translational modifications within Mascot and SEQUEST. Results were viewed and validated within Scaffold (Proteome Software Inc., Portland, OR, U.S.A; version 4.4.6), and protein identities were accepted if they could be established at >99.9% probability and contained at least one identified peptide.

Protein family identification was based on these criteria, and label free quantification of unique members of each family was performed according to normalized spectral abundance factor (NSAF) [89,90]. Percent of total proteome was then calculated for each protein family (and subgroups for SVMPs, PLA_2_s, and serine proteases).

### 5.7. Mass Spectrometry: MALDI-TOF-MS/MS

Adult and neonate venoms were electrophoresed as above, and select bands prevalent only in adult venoms (approximate masses: 7, 23, and 53 kDa) were excised and subjected to in-gel trypsin digestion. Briefly, excised bands were destained with 100% ACN in an eppendorf tube, washed with a second change of ACN, reduced and alkylated with DTT and iodoacetamide respectively, and then digested overnight with 0.6 μg Pierce Gold trypsin at 37 °C (in a total volume of 100 μL 50 mM ammonium bicarbonate buffer). Peptides generated were concentrated to 10 μl and contaminating reactants were removed via C_18_ ZipTip cleaning. One μL of cleaned sample was mixed with 1 μL of a-cyano-4-hydroxycinnamic acid (10 mg/mL in 50% ACN, 0.1% TFA). The mixture was then spotted onto the MALDI target plate, allowed to air dry and analyzed using an Ultraflex-TOF/TOF mass spectrometer (Bruker Daltonics, Billerica, MA) in positive ion, reflector mode using a 25 kV accelerating voltage (Proteomics and Metabolomics Facility, Colorado State University). Mass spectrograms and MS/MS data generated were searched using the MASCOT ver. 2.2 search engine [91,92] and the NCBI database [93]. The peptide mass tolerance was set at 0.15 Da and the peptide fragment ion mass tolerance was set at 0.8 Da. Peptides characteristic of trypsin autolysis and keratin hydrolysis were removed before analysis.

### 5.8. Venom Lethality

Toxicity of venom from one adult female (F59) and one of her offspring (F59-N1) was assayed in 25–30 g female non-Swiss albino (NSA) mice [81]. Venom doses were delivered intraperitoneally (IP) in sterile saline (100 μL bolus), with doses adjusted to individual animal body masses. Three animals per dose were utilized, and all animals were monitored for 24 h. Lethality was expressed as micrograms of venom per gram body mass (μg/g) producing 50% mortality after 24 h and was calculated from the raw mortality-dose data using the Trimmed Spearman–Karber (TSK) Program version 1.5 [94]. Animal numbers were minimized, and all procedures were approved by the University of Northern Colorado IACUC (protocol #9401). Date of approval: 11 February 2015.

### 5.9. Statistical Analyses

Venom enzymatic activities between the different age classes were analyzed with analysis of variance (ANOVA) followed by a Tukey HSD post-hoc test. Comparison of the enzymatic activity levels between adult male and adult female snakes was completed using a Student’s *t*-test. Both statistical tests were performed with R version 3.4.2. *p*-values < 0.05 were considered statistically significant. 

## Figures and Tables

**Table 1:** Relative occurrence of the different protein families present in the venom of adult *Crotalus polystictus.*

Protein Family	% of Total Venom Proteins
L-Amino Acid Oxidase	4.35
Snake Venom Metalloproteinase (SVMP)	19.53
**•** P-I SVMP	2.72
**•** P-II SVMP	8.23
**•** P-III SVMP	8.58
Phosphodiesterase (Exonuclease)	<1.0
Phospholipase A_2_	7.49
**•** Acidic PLA_2_	1.47
**•** Basic PLA_2_	6.02
Serine Protease	7.84
**•** Thrombin-like	5.69
**•** Kallikrein-like	1.90
**•** Plasminogen Activator	<1.0
Glutaminyl Cyclase	<1.0
Hyaluronidase	<1.0
Phospholipase B	<1.0
Bradykinin-Inhibitory Peptide	36.32
Cysteine-rich Secretory Protein	4.41
C-type Lectin	7.80
Vespryn	1.31
Disintegrin	9.07
Dis-Cys Fragments	1.04
Nerve Growth Factor	<1.0

## Supplementary Materials

The following are available online at http://www.mdpi.com/2072-6651/10/7/271/s1, Table S1: Full list of peptides identified following shotgun proteomic analysis of a pooled (3 adult and 3 female) *Crotalus polystictus* venom sample.
